# Long Persistent Luminescent HDPE Composites with Strontium Aluminate and Their Phosphorescence, Thermal, Mechanical, and Rheological Characteristics

**DOI:** 10.3390/ma15031142

**Published:** 2022-02-01

**Authors:** Anesh Manjaly Poulose, Hamid Shaikh, Arfat Anis, Abdullah Alhamidi, Nadavala Siva Kumar, Ahmed Yagoub Elnour, Saeed M. Al-Zahrani

**Affiliations:** 1 SABIC Polymer Research Center, Department of Chemical Engineering, King Saud University, Riyadh 11421, Saudi Arabia; hamshaikh@ksu.edu.sa (H.S.); aarfat@ksu.edu.sa (A.A.); AKFHK90@hotmail.com (A.A.); szahrani@ksu.edu.sa (S.M.A.-Z.); 2 Department of Chemical Engineering, King Saud University, Riyadh 11421, Saudi Arabia; snadavala@ksu.edu.sa (N.S.K.); aelnour@ksu.edu.sa (A.Y.E.)

**Keywords:** phosphorescent composites, thermal, mechanical, rheology

## Abstract

In this work, HDPE/strontium aluminate-based auto glowing composites (SrAl_2_O_4_: Eu, Dy (AG_1_) and Sr_4_Al_14_O_25_: Eu, Dy (AG_2_)) were prepared, and their phosphorescence studies were conducted. In HDPE/AG_1_ composites, the green emission was observed at ~500 nm after the UV excitation at 320 nm. The HDPE/AG_2_ has a blue emission at ~490 nm and, in both cases, the intensity of emission is proportional to the AG_1_ and AG_2_ content. The DSC data show that the total crystallinity of both the composites was decreased but with a more decreasing effect with the bulky AG_2_ filler. The melting and crystallization temperatures were intact, which shows the absence of any chemical modification during high shear and temperature processing. This observation is further supported by the ATR-FTIR studies where no new peaks appeared or disappeared from the HDPE peaks. The tensile strength and modulus of HDPE, HDPE/AG_1_, and HDPE/AG_2_ composites were improved with the AG_1_ and AG_2_ fillers. The rheological studies show the improvement in the complex viscosity and accordingly the storage modulus of the studied phosphorescent HDPE composites. The SEM images indicate better filler dispersion and filler–matrix adhesion, which improves the mechanical characteristics of the studied HDPE composites. The ageing studies in the glowing composites show that there is a decrease in the intensity of phosphorescence emission on exposure to drastic atmospheric conditions for a longer period and the composites become more brittle.

## 1. Introduction

The known history of persistent luminescence started at the beginning of the 17th century. In 1602, an Italian shoemaker, V. Casciarolo, observed a solid luminescence from the barite (BaSO_4_), a mineral known as the Bologna stone. The cause for the luminescent emission was unclear, but it was employed for many applications [1]. In the 20th century, the luminous paints were based on the luminescent emission from Cu- or Mn-doped zinc sulfide (green emission). Their application was later restrained by their shorter afterglow time (~30 min.) and lower brightness. These sulfide phosphors have an affinity towards moisture and CO_2_ and are chemically unstable [2]. The researchers were in search of phosphorescent material that releases visible light for a longer period even after the excitation source (UV, X-ray, etc.) has been stopped. The detection of strontium aluminate-based rare-earth-doped phosphors initiated the modern luminescent materials era. When compared with sulfide phosphors, these phosphors (e.g., SrAlxOy: Eu, Dy) exhibited better afterglow, brightness, chemical stability, environmental safety, and photo-resistance [3]. These materials gain solar light energy, remain photo-luminescent for a longer time (~16 h), and find applications in roadway displays at night-time, glowing paints, fluorescent lamps, pavements, etc. [4,5,6,7,8,9,10]. The application side of rare earth metals doped alkaline-earth metals is rapidly expanding due to the faster growth in the nanotechnology field. Due to its long persistent nature and stability, it has also been utilized in areas such as medical imaging and fluorescent probes [11,12]. 

After the invention of long-lasting SrAl_2_O_4_:Eu^2+^, Dy^3+^ phosphor in 1996, only a limited number of phosphors were developed to date, which is suitable for practical applications. In recent years, different phases of rare-earth-doped (Pr^3+^, Ce^3+^, Sm^2+^, Nd^3+^, etc.) aluminates were developed, and their emission wavelength depends upon the crystalline structure of the alkaline earth metal aluminate phase [13,14,15,16,17,18]. Among these phosphors, SrAl_2_O_4_: Eu, Dy and Sr_4_Al_14_O_25_: Eu, Dy have displayed a robust potential for phosphorescence applications and are available commercially [19,20]. These materials undergo hydrolysis in the presence of atmospheric moisture, and the encapsulation process is important to extend the glowing property and is reported in the literature [21,22,23,24,25]. Out of these methods, polymeric encapsulation attracts more due to its low cost, and the process enables the composites to be shaped easily and imparts better physical properties [26,27,28,29]. In recent years, research on polymer–phosphor material, composites have gained much attention due to their wide range of potential applications such as safety indication, emergency lighting, road signs, interior decorations, photovoltaics, and optoelectronics [30,31,32]. In one study, a facile way to synthesize a luminescent polymer nanocomposite of PMMA-(Sr_3_B_2_O_6_:Dy^3^) is presented, and detailed structural, optical, and photoluminescence properties were investigated. The nanocomposites films consisting of (Sr_3_B_2_O_6_:Dy^3^) dispersed in polymethyl methacrylate (PMMA) matrix were prepared via solution casting method [31]. In another study, the effect of incorporating different volume loadings (ratios ranging from 0.05% to 5%) of the green-emitting SrAl_2_O_4_ phosphor into both the low-density polyethylene (LDPE) matrix and the (PMMA) matrix was investigated. The composites were produced by the melt-mixing process, and the results showed that LDPE can be used to build a suitable three-dimensional phosphor network for luminescence applications [33]. In recent studies, the incorporation of SrAl_2_O_4_:Eu^2+^, Dy^3+^ powder into polylactic acid (PLA) matrix, as an example of biodegradable polymers with good thermo-plasticity and machinability, was investigated. The results showed that the concentration of SrAl_2_O_4_:Eu^2+^, Dy^3+^ significantly impacts the fluorescence and mechanical properties of resulting composites and that the composites with 15 wt.% have the best fluorescence properties [34]. Furthermore, the modification of the SrAl_2_O_4_:Eu^2+^, Dy^3+^ powder with SiO_2_ has brought about an improved filler dispersion and compatibility with the (PLA) matrix for 3D printing technologies [26]. 

Due to its excellent mechanical properties and ease of manufacturing, high-density polyethylene (HDPE) is recognized as one of the most versatile commodity thermoplastics, and it finds a wide acceptance in many industrial applications. The unique properties of HDPE combined with the attractive luminescent features will widen the window of its application in several other interesting areas such as nighttime display boards, sensing, etc. Furthermore, in these reported works the phosphorescence ageing studies exposing the luminescence film to drastic outside temperature conditions are missing. Therefore, in this work, two different strontium aluminate-based phosphorescent materials were melt-mixed with HDPE matrix, and adding to their luminescence, thermal, mechanical, rheological, and morphological studies, the phosphorescence ageing studies were also conducted. The solution casting method makes use of strong solvents, which will inversely affect the mechanical properties of the resultant composites and hence the melt-mixing process is preferred in the present study. 

## 2. Materials and Methods

### 2.1. Materials

High-density polyethylene (TASNEE HD F0455) was supplied by TASNEE, Jubail Industrial City, Saudi Arabia, having a density of 0.957 g/cm^3^ (ISO 1183). It has a melt flow index (190 °C/5 kg) of 0.40 g/10 min. (ISO 1133). Two strontium aluminate-based phosphors (SrAl_2_O_4_: Eu, Dy (Mw = 209.11 g/mol) (AG_1_) and Sr_4_Al_14_O_25_: Eu, Dy (1139.55 g/mol) (AG_2_), were bought from Sigma Aldrich Company, St. Louis, MO, USA.

### 2.2. Methods

#### 2.2.1. Composites Preparation

The strontium aluminate phosphors AG_1_ and AG_2_ (1, 3, 5, and 10 wt.%) were melt-blended with the HDPE matrix in DSM Xplore micro-compounder, Sittard, Netherlands, at a temperature of 200 °C at 100 rpm for a mixing time of 3 min. Thin films with an average thickness of 0.5 mm were prepared with the help of COLLIN Press, Maitenbeth, Germany (100 bar pressure, 200 °C), for the phosphorescence measurements. Dumb-bell-shaped tensile testing specimens ASTM, Type1, were prepared with a microinjection molding machine, DSM Xplore, Sittard, Netherlands (12 cm^3^). The dumb-bell specimens were shaped in a mold maintained at 6 bar pressure and at room temperature. 

#### 2.2.2. Composites Characterization

The thermal analysis of the samples (DSC) was carried out in Shimadzu DSC-60A, Kyoto, Japan, taking 5–10 mg material in an aluminum pan, and the temperature program was from 30 to 220 °C at 10 °C/min heating and cooling rate with 4 min. holding time in the melt. 

The crystallinity percent was obtained as:

$$X_{c}\%=\frac{\Delta H_{m}}{\left(1-\Phi \right)\Delta H_{m}^{{}^{\circ}}}$$

where (Φ) is the weight fraction of filler in the composites, ($\Delta H_{m}$) is the enthalpy of melting obtained from the DSC melting peak, and ($\Delta H_{m}^{{}^{\circ}}$) is the theoretical value of 100% crystalline HDPE, which is 293 J/g [35]. 

The chemical composition of prepared composites was investigated in an Attenuated Total Reflection-Fourier Transform Infrared Spectroscopy (ATR-FTIR), from Thermo-Scientific, Waltham, MA USA, Nicolet iN10 model with a germanium micro-tip. The FTIR scanning range was between 400–4000 cm^−1^.

The morphology and dispersion of the filler particles were studied using SEM. A scanning Electron Microscope (SEM), JEOL JSM-6360A, (Tokyo, Japan), at an accelerating voltage of 20 kV, was used for these analyses. The samples for SEM examination were cryo-fractured after exposure to liquid nitrogen. The elemental detection was carried out with the help of the attached energy-dispersive X-ray spectroscopy (EDS) facility.

Tensile testing was carried out in a Hounsfield H100 KS (Salfords, UK) universal testing machine at a crosshead speed of 10 mm/min according to the ASTM D638 standard testing method, and an average of five tests is reported.

The rheological properties of HDPE/AG_1_ and HDPE/AG_2_ samples were carried out in a TA instruments, New Castle, DE, USA, ARG2 model, with parallel-plate geometry. The experimental temperature was set at 190 °C so that the composites were in a melt state, and the gap between the parallel plate was kept at 1000 μm for the analysis. The angular frequency sweep tests were performed from 0.01 to 628.3 rad/s under an oscillation of 3.259 Pa.

Phosphorescence measurements were performed on an Agilent Technologies Fluorescence Spectrophotometer, Santa Clara, CA, USA equipped with a Xe lamp as a source of UV. The wavelength of excitation used was 320 nm, and the emission spectra from these composites were monitored in the visible range.

#### 2.2.3. Phosphorescence Intensity vs. Time in HDPE/AG_1_ and HDPE/AG_2_ Composites

The phosphorescence decay in these composites was carried out in an Agilent Technologies Fluorescence Spectrophotometer with the delay time and gate time as 0.1 and 10,000 ms. The excitation and emission wavelength is chosen were 320 and 490 nm, respectively, and the decay in the intensity of emission was monitored for a period of 1400s. 

#### 2.2.4. Effect of Ageing on Phosphorescence and Mechanical Properties

HDPE/3AG_1_ composites films and standard dumb-bell specimens were chosen for the ageing studies (tensile and phosphorescence) and were exposed to outside climatic conditions for 40 days (August-September; Riyadh, Saudi Arabia). The measurements of phosphorescence emission intensity and mechanical properties were monitored in a time interval of 10 days. The ageing phenomenon is mainly influenced by the exposed temperature and humidity. The average day and nighttime temperatures for every 10 days were 42.9, 43.1, 41.6, and 38.4 °C and 28.3, 27.6, 27.6, and 24.5 °C; respectively. The average humidity value was around 10% during that period.

## 3. Results and Discussion

### 3.1. Particles and Composites SEM Analysis

**Figure 1:**
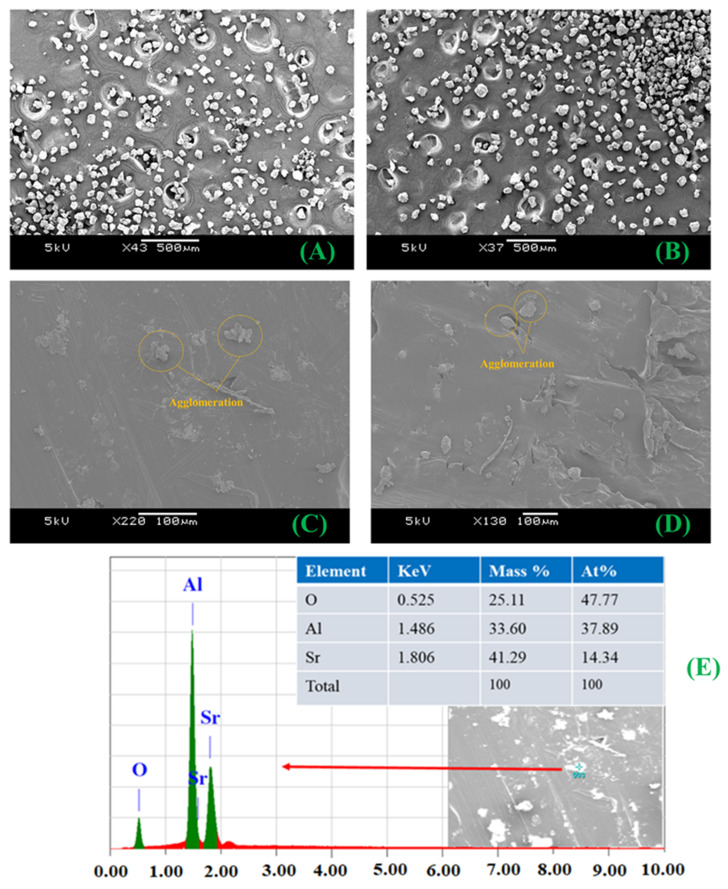
SEM images of AG_1_ (**A**) and AG_2_ (**B**) powders; and HDPE/10AG_1_ (**C**), HDPE/10AG_2_ (**D**), and EDS of HDPE/10AG_1_ (**E**) composites.

The SEM analysis of AG_1_ and AG_2_ powders are shown in Figure 1A,B, respectively. While that of HDPE/10AG_1_ and HDPE/10AG_2_ composites along with the EDS elemental analysis are shown in Figure 1C–E, respectively. From Figure 1A,B, the SEM images of powder samples, it can be seen that both samples have irregular (random) particle shapes with particle sizes ranging between 5 to 150 µm approximately.

Though agglomeration is visible on the SEM images in the highest filler loading composites (Figure 1C,D), the dispersion of the filler and the adhesion among fillers and HDPE is good enough to improve the tensile and storage modulus of the studied composites. The EDS analysis is carried out on the particle visible on the SEM images, and the analysis shows the presence of elements such as Sr, Al, and O_2_, confirming the composition of the AG_1_ fillers, as in Figure 1E.

### 3.2. DSC and FTIR Data for HDPE/AG_1_ and HDPE/AG_2_ Composites

**Figure 2:**
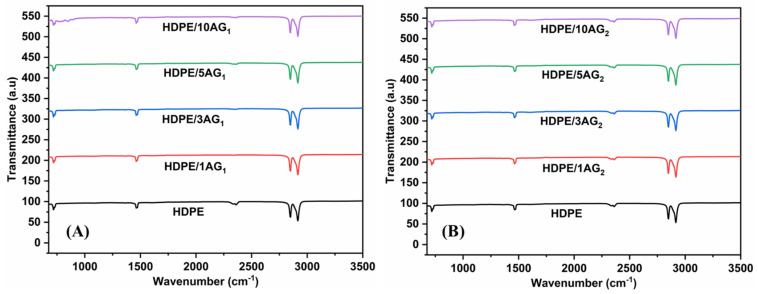
ATR-FTIR data of HDPE and HDPE/AG_1_ (**A**) and HDPE/AG_2_ (**B**) composites.

The DSC results for the HDPE matrix and the composites are shown in Table 1 and Table 2. The AG_1_ and AG_2_ filler do not affect the melting temperature of HDPE, showing that the mixing process is purely physical, and this observation is also supported by the ATR-FTIR data. As shown in Figure 2A,B, the HDPE and the composites with AG_1_ and AG_2_ have similar FTIR peaks, confirming the absence of any chemical modification of HDPE during the high-temperature mixing process. 

On the other hand, the AG_1_ and AG_2_ fillers have a significant effect on the crystallinity of HDPE, as shown in Table 1 and Table 2, with a more pronounced effect on composites with AG_2_ filler due to the bulky chemical structure of AG_2_ than AG_1_ [36].

### 3.3. Mechanical Characterization of HDPE/AG_1_ and HDPE/AG_2_

**Figure 3:**
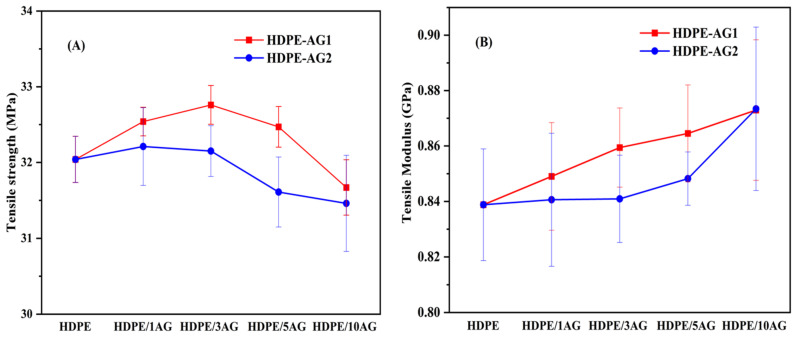
Tensile strength (**A**) and tensile modulus (**B**) in HDPE, HDPE/AG_1_, and HDPE/AG_2_ composites.

The mechanical properties of the composites are important for the application side of the auto-glowing composites. The tensile strength and modulus of HDPE, HDPE/AG_1_, and HDPE/AG_2_ composites are shown in Figure 3A,B, respectively. It is found that at lower filler loading (1–3 wt.%), the tensile strength was found to be improved and then decreases on further filler loading due to the agglomeration of the fillers, especially at higher filler loading concentration. The tensile modulus increases from 0.84 to 0.873 GPa, increasing the filler content from 0 to 10 wt.%. 

### 3.4. Rheological Studies in HDPE/AG_1_ and HDPE/AG_2_ Composites

**Figure 4:**
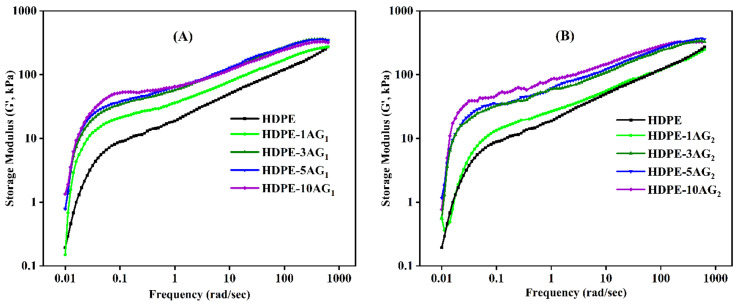
Storage modulus in HDPE/AG_1_ (**A**) and HDPE/AG_2_ (**B**) composites.

**Figure 5:**
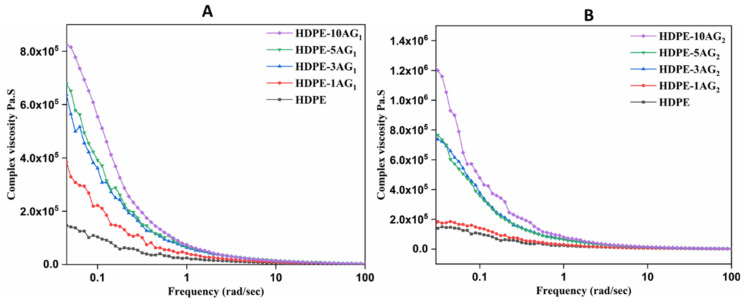
Complex viscosity data in HDPE/AG_1_ (**A**) and HDPE/AG_2_ (**B**) composites.

The storage modulus and complex viscosity of HDPE/AG_1_ and HDPE/AG_2_ composites are shown in Figure 4 and Figure 5, respectively. For both composites, the AG_1_ and AG_2_ fillers have a positive effect on both the complex viscosity and storage moduli of the composites, especially at a lower frequency range. The complex viscosity increment shows the good filler–HDPE interaction and improves the storage moduli of the studied composites. As shown in Figure 4, there is a gradual increase in the moduli value of the composites with the AG_1_ and AG_2_ content. 

### 3.5. Phosphorescence Studies in HDPE/AG_1_ and HDPE/AG_2_ Composites

The physical mechanism behind the phosphorescence phenomena is briefly divided into four processes: (i) when the phosphor is excited by external excitation illumination at specific wavelengths takes place upon the liberation of charge carriers (electrons and/or holes); (ii) the excited electrons or generated holes can be non-radiatively acquired by the electron or hole traps through a conduction band (CB) or valence band (VB), respectively, or by the quantum tunneling process through the forbidden band, known as the trapping process. The traps do not emit electromagnetic radiation but they store the excitation energy for a long time (optical battery). (iii) After stopping the excitation, the captured charge carriers can be released mainly by thermal stimulation energy (other stimulations like optical or mechano-ones are out of the scope in this paper), termed a de-trapping process. (iv) Finally, the released charge carriers move back to the emission center, yielding the delayed luminescence due to the electron–hole recombination [37,38,39].

**Figure 6:**
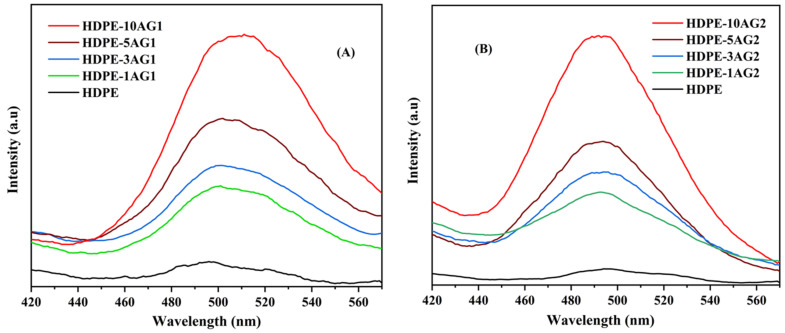
Phosphorescence emission in HDPE, HDPE/AG_1_ (**A**), and HDPE/AG_2_ (**B**) composites.

The phosphorescence measurements for HDPE/AG_1_ and HDPE/AG_2_ are shown in Figure 6A,B, respectively. The UV excitation was carried out at 320 nm, and the intensity of emission was monitored in HDPE composites with AG_1_ and AG_2_ fillers. In HDPE/AG_1_ composites, green emission was observed at ~500 nm, due to the 4f^6^5d^1^ to 4f^7^ transition of Eu^+2^ ion of the AG_1_ phosphor sample, and, as expected, the intensity of emission was found to increase with the increased loading percentage of the AG_1_ phosphor, as shown in Figure 6A. For the HDPE/AG_2_, a blue emission at ~490 nm was observed, and similar to the AG_1_ sample, the emission intensity is proportional to the AG_2_ content (Figure 6B).

**Figure 7:**
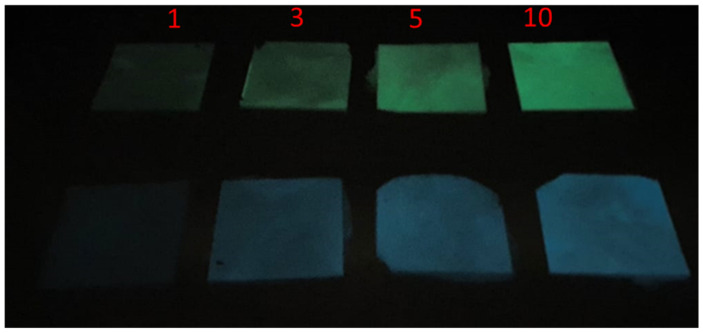
Phosphorescence emission under darkness; green for AG_1_ composites and blue for AG_2_ composites (1–10 wt.%).

The HDPE/AG_1_ (green) and HDPE/AG_2_ (blue) emission under darkness can be visible from Figure 7, and the intensity of emission is proportional to the filler content (AG_1_ and AG_2_: 1 to 10 wt.%).

### 3.6. Phosphorescence Intensity Decay Studies in HDPE/AG_1_ and HDPE/AG_2_ Composites

**Figure 8:**
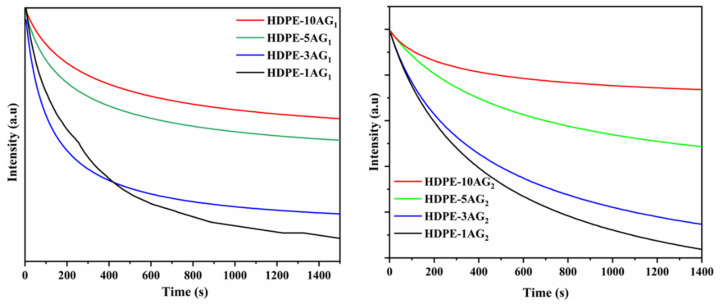
Phosphorescence intensity vs. time in HDPE/AG_1_ and HDPE/AG_2_ composites.

The phosphorescence decay for the studied composites is shown in Figure 8. In both composites, the phosphorescence intensity decay rate is inversely proportional to the AG_1_ and AG_2_ filler content, i.e., the composites with higher filler content decay slowly. One can see that, with the 10 and 5 wt.% AG_1_ and AG_2_ composites, the decay rate is very slow and the final intensity is not reaching a zero value as previously reported for similar materials [34,40]. The afterglow for these materials is considerably higher; therefore, no such behavior was observed within the studied timespan. 

### 3.7. Effect of Ageing on Phosphorescence and Mechanical Properties

**Figure 9:**
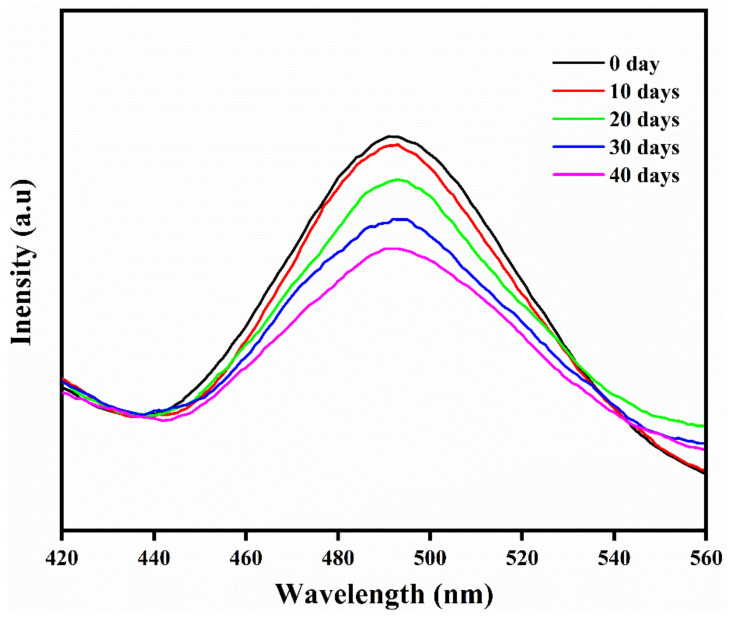
Phosphorescence decay for HDPE/3AG_1_ composites every 10 days.

The effect of aging on the phosphorescence and mechanical properties of the composite samples were conducted by the composite films to outside atmospheric conditions, for different exposure times (up to 40 days), and the phosphorescent and mechanical properties measurements were conducted in 10 days. For the sake of brevity, only the HDPE/3AG_1_ composite sample was selected. Considering the phosphorescence characteristics of composites, it was found that there is a decrease in the emission intensity due to the ageing phenomena and that this decrease in intensity is directly proportional to the number of exposure days (Figure 9). On the other hand, the effect of aging on tensile strength was found to be negligible, and the tensile modulus values decrease on ageing. It was also observed that both tested samples, neat HDPE and HDPE/3AG_1_ composite, became more brittle, as evidenced by the reduction in elongation percentages with an increased number of exposure days. The elongation at break for neat HDPE was found to be decreased from 58.5 to 18.82, while that of HDPE/3AG_1_ composite was from 48 to 11 (Table 3). This latter observation is due to the physical aging phenomena that takes place for the sample at the outside atmospheric conditions [41].

## 4. Conclusions

Long afterglow HDPE-based strontium aluminate composites were prepared and characterized for their application side. The HDPE-encapsulated AG_1_ and AG_2_ composites resulted in long afterglow composites, which lasted for many hours. The physical mixing was confirmed from both the DSC and ATR-FTIR studies. The green (~500 nm) and blue emission (~490 nm) were observed for the AG_1_ and AG_2_ composites, respectively, and the intensity of emission improves with the amount of AG_1_ and AG_2_ fillers. The DSC data show that the total crystallinity of both the composites was decreased but with a more decreasing effect with the bulky AG_2_ filler without affecting the melting and crystallization temperature. The rheological results show the increase in complex viscosity and thereby the storage modulus values in the resulted composites. SEM pictures reveal good dispersion of the fillers in the HDPE matrix, and the tensile strength and modulus were found to be increased with the proportion of the fillers. Due to the better mechanical characteristics and long afterglow time, these composites can be found in applications in roadway nighttime displays, fluorescent lamps, etc. The ageing studies in the glowing composites show that there is a decrease in the intensity of phosphorescence emission on exposure to outside temperature for a longer period, and the composites become more and more brittle on ageing.

## Figures and Tables

**Table 1:** DSC results on HDPE and HDPE/AG_1_ composites.

Material	*T*_c_ (°C)	*T*_m_ (°C)	Δ*H*_m_ (J/g)	*X*_c_ (%)
HDPE	111.1	134.4	190.2	64.9
HDPE/1AG_1_	110.4	135.0	181.4	61.9
HDPE/3AG_1_	111.4	134.1	173.6	59.2
HDPE/5AG_1_	110.2	134.6	172.1	58.7
HDPE/10AG_1_	110.0	135.0	163.1	55.7

**Table 2:** DSC results on HDPE and HDPE/AG_2_ composites.

Material	*T*_c_ (°C)	*T*_m_ (°C)	Δ*H*_m_ (J/g)	*X*_c_ (%)
HDPE	111.1	134.4	190.2	64.9
HDPE/1AG_2_	111.5	134.3	174.1	59.4
HDPE/3AG_2_	110.1	135.4	160.4	54.7
HDPE/5AG_2_	110.5	134.5	160.0	54.6
HDPE/10AG_2_	110.6	134.3	154.1	52.6

**Table 3:** Tensile strength, tensile modulus, and elongation data on HDPE and HDPE/3AG_1_ composites on ageing.

Material	Tensile Strength MPa	SD	Tensile Modulus GPa	Elongation %	SD
HDPE	32.04	0.31	0.84	58.5	4.58
HDPE-10days	33.63	0.49	0.70	51.22	5.17
HDPE/20days	34.12	1.48	0.67	39.51	6.31
HDPE/30days	33.51	0.20	0.66	20.28	7.5
HDPE/40 days	32.1	0.63	0.64	18.82	3.1
HDPE/3AG_1_	31.94	0.34	0.84	48.14	4.11
HDPE/3AG_1_/10days	34.41	0.32	0.82	40.12	3.12
HDPE/3AG_1_/20days	34.12	0.51	0.79	36.32	4.2
HDPE/3AG_1_/30days	33.51	0.27	0.77	15.66	3.02
HDPE/3AG_1_/40days	33.95	0.59	0.72	11.21	2.51

## Data Availability

The data presented in this study are available on request from the corresponding author.
